# *Bacillus subtilis* natto: a non-toxic source of poly-γ-glutamic acid that could be used as a cryoprotectant for probiotic bacteria

**DOI:** 10.1186/2191-0855-3-36

**Published:** 2013-07-05

**Authors:** Aditya R Bhat, Victor U Irorere, Terry Bartlett, David Hill, Gopal Kedia, Mark R Morris, Dimitris Charalampopoulos, Iza Radecka

**Affiliations:** 1 University of Wolverhampton, Wolverhampton, UK; 2 University of Reading, Reading, UK

**Keywords:** Probiotics, γ-PGA, Cryoprotectant, Toxicity, *Bifidobacteria*, *Lactobacillus*

## Abstract

It is common practice to freeze dry probiotic bacteria to improve their shelf life. However, the freeze drying process itself can be detrimental to their viability. The viability of probiotics could be maintained if they are administered within a microbially produced biodegradable polymer - poly-γ-glutamic acid (γ-PGA) - matrix. Although the antifreeze activity of γ-PGA is well known, it has not been used for maintaining the viability of probiotic bacteria during freeze drying. The aim of this study was to test the effect of γ-PGA (produced by *B. subtilis* natto ATCC 15245) on the viability of probiotic bacteria during freeze drying and to test the toxigenic potential of *B. subtilis* natto. 10% γ-PGA was found to protect *Lactobacillus paracasei* significantly better than 10% sucrose, whereas it showed comparable cryoprotectant activity to sucrose when it was used to protect *Bifidobacterium breve* and *Bifidobacterium longum*. Although γ-PGA is known to be non-toxic, it is crucial to ascertain the toxigenic potential of its source, *B. subtilis* natto. Presence of six genes that are known to encode for toxins were investigated: three component hemolysin (*hbl D/A*), three component non-haemolytic enterotoxin (*nheB*), *B. cereus* enterotoxin T (*bceT*), enterotoxin FM (*entFM*), sphingomyelinase (*sph*) and phosphatidylcholine-specific phospholipase (*piplc*). From our investigations, none of these six genes were present in *B. subtilis* natto. Moreover, haemolytic and lecithinase activities were found to be absent. Our work contributes a biodegradable polymer from a non-toxic source for the cryoprotection of probiotic bacteria, thus improving their survival during the manufacturing process.

## Introduction

Over the years, extensive research has been done to determine the efficacy of probiotic foods in controlling and alleviating disorders/diseases (de Moreno de LeBlanc et al. 2007 Falagas et al. 2007 Garrait et al. 2009 Lara-Villoslada et al. 2007). Probiotic bacteria are helpful in maintaining a healthy gut and have been used for controlling several types of gastrointestinal infections (Anukam et al. 2008 Benchimol and Mack 2004 Kligler et al. 2007 Lara-Villoslada et al. 2007 Park et al. 2007 Pochapin 2000 Szymanski et al. 2006). Some lactic acid bacteria have been shown to have antitumor activity (de LeBlanc et al. 2005). Research has also shown a marked reduction in total serum cholesterol in human volunteers after ingestion of *Enterococcus faecium* (EF) M-74 enriched with selenium (Hlivak et al. 2005). Furthermore, strains of *Lactobacillus* and *Bifidobacterium* have been shown to cure dental disorders (Allaker and Douglas 2009). Because of these beneficial effects, probiotic microorganisms have been introduced into a variety of food and drink products for administration to humans or animals. Various strains of *Lactobacillus* and *Bifidobacterium* are used commonly as probiotic bacteria to benefit the health of the host (Benno and Mitsuoka 1992 de LeBlanc et al. 2005 De Simone et al. 1992 Kailasapathy and Rybka 1997).

One of the most important manufacturing steps for producing a probiotic food product is to introduce the bacteria into the foodstuff as dry cultures. Working with dry cultures is advantageous since they are easier to handle and have a longer shelf life than wet cultures. Freeze drying has been used most commonly for producing dry bacterial powders. However, the methods for preparation of freeze dried probiotic bacteria are often detrimental to the cell structure and viability (Saarela et al. 2005). Previously, it has been shown that the viability of lactic acid bacteria reduces by 3 log CFU/g when freeze dried (Jagannath et al. 2010). Therefore, there remains a need to improve the viability of probiotic microorganisms as they pass through, in particular, the freeze drying process.

This research uses bacterial poly-γ-glutamic acid (γ-PGA) for protecting probiotic bacteria during freeze drying. γ-PGA is a non-toxic, non-immunogenic and biodegradable biopolymer that is produced by bacteria for use in various applications. γ-PGA is edible and is present in a traditional Japanese dish Natto which is made by fermenting soyabean with Bacillus strains. Most commonly, strains of *Bacillus subtilis* and *Bacillus licheniformis* have been researched for its production (Buescher and Margaritis 2007 Candela and Fouet 2006 Shih and Van 2001). Although the antifreeze properties of γ-PGA have been determined (Mitsuiki et al. 1998 Shih et al. 2003), it has never been used to protect live probiotic bacteria during freeze drying. This research was designed to analyse the effect of different concentrations of γ-PGA as a cryoprotectant for probiotic bacteria during freeze drying. The cryoprotective effect of the polymer produced by *B. subtilis* natto was investigated by assessing the viability of several probiotic bacteria (*Lactobacillus paracasei*, *Bifidobacterium breve* and *Bifidobacterium longum*) during freeze drying.

Whilst γ-PGA is suggested to be non-toxic and non-immunogenic (Bajaj and Singhal 2011), it is important to ascertain the toxigenic potential of the specific bacterium used to produce the polymer for a novel food application (SCAN 2000). Consequently, *B. subtilis* natto was also screened for genes encoding toxins in other members of *Bacillus* sp. (Matarante et al. 2004). The presence/absence of haemolytic and lecithinase activities in this bacterium was also determined. Therefore, this research not only assesses the potential of γ-PGA as a cryoprotectant for probiotic bacteria, but also ascertains the toxigenic potential of its source, *B. subtilis* natto.

## Material and methods

### Poly- γ-glutamic acid (γ-PGA)

γ-PGA (MW: 257,000 Da) was produced and extracted from *B. subtilis* natto ATCC 15245 and identified by Fourier transform infrared (FTIR) spectroscopy as reported earlier (Kedia et al. 2010).

#### Bacterial strains

Three probiotic bacteria (*Bifidobacterium longum* NCIMB 8809, *Bifidobacterium breve* NCIMB 8807 and *Lactobacillus paracasei* NCIMB 8835) were obtained from National Collection of Industrial and Marine Bacteria (NCIMB), Aberdeen, UK. The stock cultures were freeze-dried and stored at −20°C. Before use, the cultures were revived aseptically and grown anaerobically on Bifidobacteria Selective Medium (BSM) Agar for *Bifidobacteria* and MRS agar for *Lactobacillus* at 37°C in anaerobic gas jars using an atmosphere generation system (Oxoid Anaerogen™, UK) and indicator strip according to manufacturer’s instructions. Both BSM and MRS broth/agar were purchased from Sigma-Aldrich, U.K. and prepared according to the manufacturer’s protocol.

#### Growth media

BSM agar/broth were used for the growth and enumeration of Bifidobacteria under study (Nualkaekul et al. 2011) and MRS medium was used for *Lactobacillus*.

#### γ-PGA as a cryoprotectant

Initially, unsterilised γ-PGA was used for preliminary cryoprotection experiment. However, due to potential concerns on the safety of future products, γ-PGA was sterilised by autoclaving at 110°C and 0.35 bar for 30 min before being used for cryoprotection studies. The structural integrity of the polymer was determined by comparing the FTIR spectra (Genesis II FTIR™, UK) of the polymer before and after sterilization.

To prepare cells for freeze drying, all microorganisms were cultured anaerobically at 37°C. *B. breve* and *B. longum* were cultured in 250 ml of BSM broth for 22 h and 16 h respectively, while *L. paracasei* was cultured in 250 ml of MRS broth for 48 h. After incubation, viable counts were performed on BSM agar (*Bifidobacteria*) and MRS agar (*Lactobacillus*) to determine the number of viable cells prior to freeze drying. The cultures were centrifuged and washed with PBS to obtain cell pellets and then resuspended in 10 ml solutions of either 10% (w/v) γ-PGA, 5% (w/v) γ-PGA or 10% (w/v) sucrose. For cells without a cryoprotectant, 10 ml of sterile distilled water was added. The suspensions were incubated at room temperature for 1 h and then frozen at −80°C for 24 h. The frozen cultures were then freeze dried (Edwards Freeze Dryer Modulyo) at −40°C and 5 mbar for 48 h. After freeze drying, 10 ml of PBS was added to each treatment and the viability was determined. Cells were enumerated by the Miles and Misra technique which involves a 10 fold dilution series in PBS followed by aseptically plating out 20 μl of each cell suspension in triplicate on appropriate media, which were then incubated anaerobically at 37°C.

#### Scanning electron microscopy (SEM) analysis

SEM analysis was performed to determine the surface structure of freeze dried cells protected with γ-PGA. All freeze dried samples were coated with gold using a sputter coater (Emscope Sc 500). SEM analysis was performed using a Zeiss EVO50 and the images analysed using the Smart SEM software.

### Toxigenic analysis

The presence of major enterotoxins and virulence factors in *B. subtilis* natto was investigated and compared to *B. cereus* which was used as a positive control (Matarante et al. 2004). The genes encoding various enterotoxins and enzymes screened were: HBL - a three component hemolysin (*hbl D/A*), NHE - three component non-haemolytic enterotoxin (*nheB*), *B. cereus* enterotoxin T (bceT), enterotoxin FM (*entFM*), sphingomyelinase (*sph*), phosphatidylinositol and phosphatidylcholine specific phospholipase (*piplc*). Phosphofructokinase A (*pfkA*), a housekeeping gene, was used as positive control.

#### DNA isolation and purification

5 ml of Tryptone Soya Broth was inoculated with a single colony of *B. subtilis* natto or *B. cereus* and the cultures were incubated at 30°C overnight with shaking at 150 rpm. Bacterial cells were harvested by centrifugation at 1,000 × g for 5 min and the bacterial pellets were washed with sterile water. DNA was isolated using the GenElute Bacterial Genomic DNA kit (Sigma-Aldrich, UK) according to the protocol provided by the manufacturer. The DNA quantity was determined using a Nanodrop2000 Spectrophotometer (Thermo Scientific, UK).

#### PCR analysis

The primers for PCR analysis were purchased from Sigma-Aldrich, UK. The details of the primers used for screening the genes of interest are provided in Table 1. The gene sequence of *pfkA* was obtained from the nucleotide collection of the National Centre for Biotechnology Information (NCBI) and the primers for the gene were designed using the NCBI Primer-Blast. PCR analysis was performed on isolated and purified DNA from each microorganism as described by (Matarante et al. 2004). Amplification comprised of an initial denaturation at 94°C for 3 min followed by 31 cycles of denaturation (94°C for 30 s), annealing (58°C for 45 s) and extension (72°C for 1 min 30 s). A final extension at 72°C for 5 min was also performed. Control mixtures without template DNA were also included in each experiment. PCR amplification was carried out in an MJ Research PTC-200 Peltier Thermal Cycler. *Taq* polymerase, deoxynucleotide triphosphates and DNA molecular weight markers were purchased from Roche, UK. The amplified fragments were separated by electrophoresis on a 2% agarose gel.

**Table 1:** **PCR primers used and virulence factors (adapted from Matarante et al.**^2004^**)**

**Target gene**	**Primer name**	**Primer sequence (5′-3′)**	**Amplicon size (bp)**	**Reference**
*hbl – D/A*	hblD-f	GGAGCGGTCGTTATTGTTGT	623	(Matarante et al. 2004)
	hblA-r	GCCGTATCTCCATTGTTCGT		
*nheB*	nheB 1500 S	CTATCAGCACTTATGGCAG	769	(Matarante et al. 2004)
	nhe B 2269 A	ACTCCTAGCGGTGTTCC		
*bceT*	ETF	TTACATTACCAGGACGTGCTT	428	(Matarante et al. 2004)
	ETR	TGTTTGTGATTGTAATTCAGG		
*entFM*	EntA	ATGAAAAAAGTAATTTGCAGG	1269	(Matarante et al. 2004)
	EntB	TTAGTATGCTTTTGTGTAACC		
*sph*	Ph1	CGTGCCGATTTAATTGGGGC	558	(Matarante et al. 2004)
	Ph2	CAATGTTTTAAACATGGATGCG		
*piplc*	PC105	CGCTATCAATGGACCATGG	569	(Matarante et al. 2004)
	PC106	GGACTATTCCATGCTGTACC		
pfkA*	pfkA-F	CCATCAGCTAAACCAGCC	370	This study
	pfkA-R	CGCGGTGGTACGAAATTA		

* *pfkA* primer was designed using the NCBI Primer-Blast.

### Determination of haemolytic and lecithinase activities

Haemolytic and lecithinase activies were determined using a method described previously (Matarante et al. 2004). Haemolytic activity was determined at 30°C on blood agar plates containing 5% horse blood. Lecithinase (phosphatidylinositol-specific phospholipase C) activity was determined at 30°C on nutrient agar supplemented with 8% egg yolk emulsion. *B. cereus* was used as a positive control. 5 μl of overnight TSB cultures of both microorganisms incubated at 37°C were used to inoculate each of the media plates in triplicate. The cell-free supernatants of each microorganism were also tested for their haemolytic and lecithinase activities by inoculating 5 μl of each cell free supernatants into the aforementioned media (Matarante et al. 2004).

### Statistical analyses

All results were statistically analysed using Microsoft Excel 2010 and GraphPad Prism 5. Two-factor Anova and student’s *T* test were used to compare data. The Bonferroni multiple comparison test was used for the non-parametric analysis of the data to determine the difference between individual groups in a data set. P value ≤ 0.05 was considered to be statistically significant.

## Results

### γ-PGA as a cryoprotectant for probiotic bacteria

**Figure 1:**
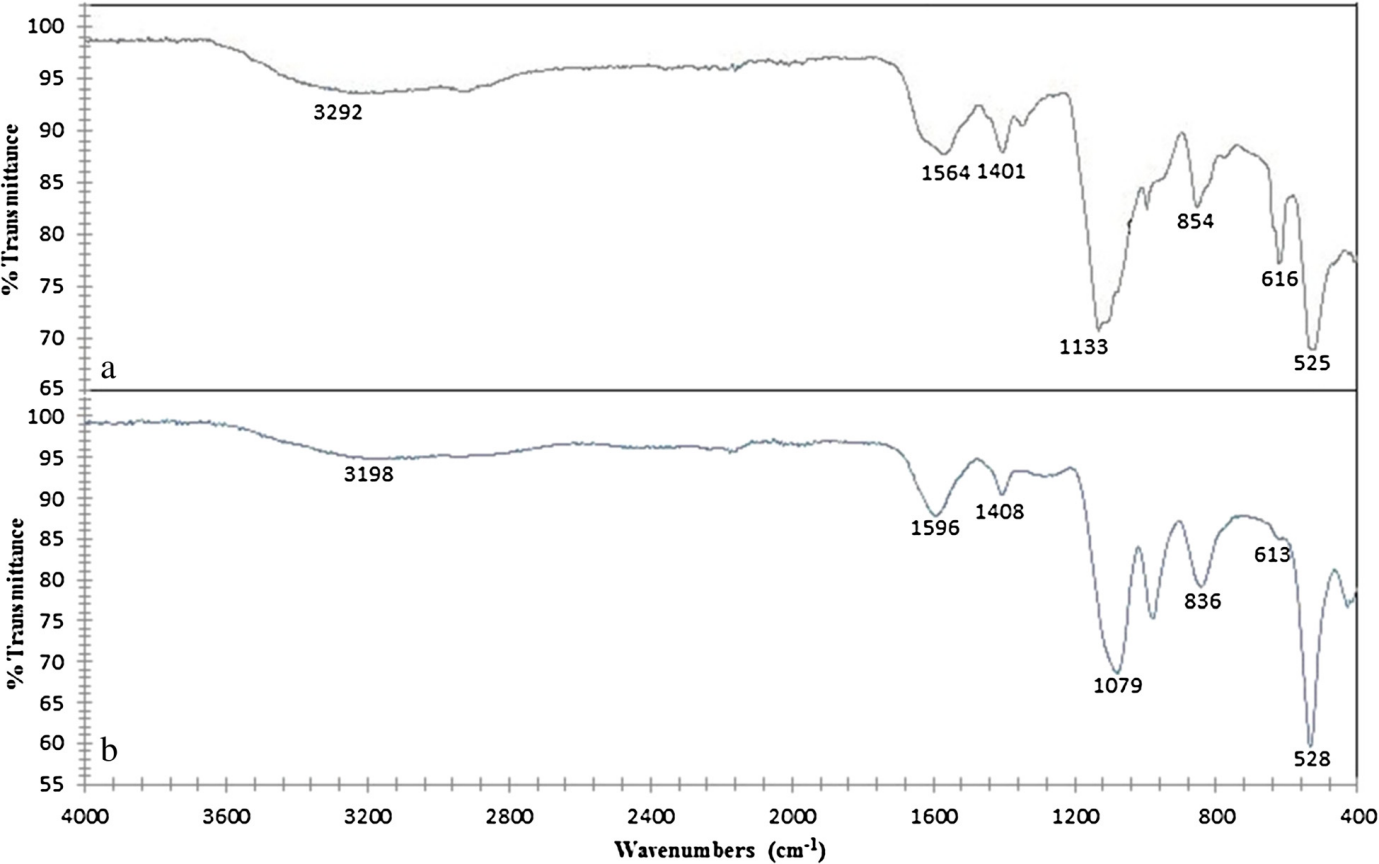
FT-IR Spectroscopy of (a) unsterilised γ-PGA and (b) sterilised γ-PGA showing relevant peaks.

The effect of sterilization on γ-PGA was investigated using FTIR to determine whether steam sterilization alters the structure of the polymer. Results from FTIR spectroscopy (Figure 1) indicated that the FTIR spectra for sterilised and unsterilised polymer showed all the peaks representing the characteristic bonds in γ-PGA. This indicates that steam sterilisation does not alter the chemical integrity of the polymer.

**Figure 2:**
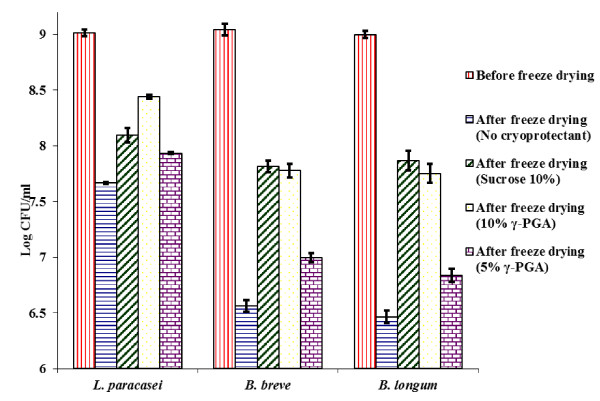
**Effect of γ-PGA and sucrose on viability of probiotic bacteria during freeze drying.** Cells were freeze dried at −40°C and 5 mbar pressure and viability was measured before and after freeze drying on BSM agar. Experiments were conducted in triplicate (n = 3).

10% γ-PGA, 5% γ-PGA and 10% sucrose were used to protect probiotic bacteria during freeze drying and the effect of cryoprotection was assessed (Figure 2). It was observed that when no cryoprotectant was used to protect the cells, *L. paracasei* showed a reduction in viability of 1.34 log CFU/ml. When 10% sucrose was used to protect *L. paracasei* during freeze drying, 0.91 log CFU/ml reduction in viability was observed after freeze drying. However, for 10% γ-PGA-protected cells, the loss in viability was reduced to 0.51 log CFU/ml. For *L. paracasei*, 10% γ-PGA was able to protect the cells significantly (P ≤ 0.05) better than 10% sucrose. The cryoprotectant ability of 5% γ-PGA (Figure 2) was comparable to 10% sucrose (P > 0.05). A more pronounced reduction in viability (~2.5 log CFU/ml) was observed when both *Bifidobacterium* strains were freeze dried without any cryoprotectant (Figure 2). When the cells were protected with 10% γ-PGA, only 1.24 - 1.26 log CFU/ml reduction in viability was observed. The cryoprotectant ability of 10% sucrose and 10% γ-PGA for the *Bifidobacterium* strains was comparable (P ≥ 0.05).

### SEM analysis

**Figure 3:**
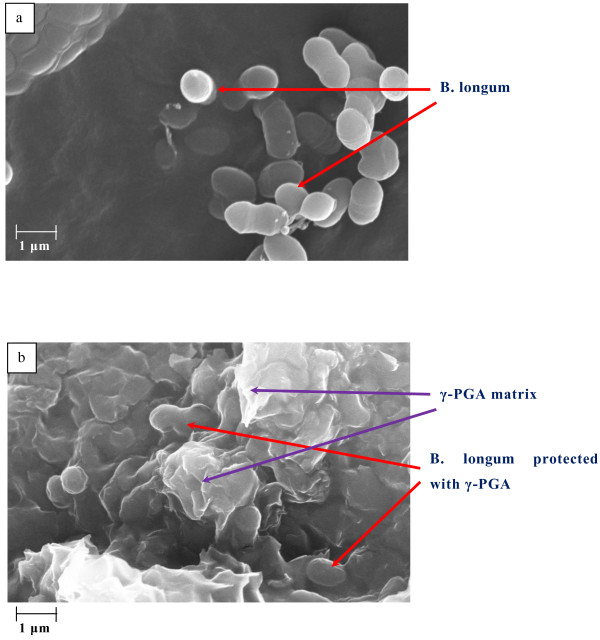
**SEM image of a) Freeze dried *B. longum *cells with no γ-PGA protection (EHT = 20.00 kV; Signal A = SE1; WD = 4.0 mm) b) Freeze dried *B. longum *protected with γ-PGA (EHT = 20.00 kV; Signal A = SE1; WD = 4.5 mm).** SEM analysis was performed using Zeiss EVO50, U.K. and photographs were analysed using the software provided by Zeiss EVO50.

Freeze dried bifidobacteria with and without γ-PGA as a cryoprotectant were analysed using SEM to understand how the cells may be protected. As is evident from Figure 3a and b, freeze dried *B. longum* cells protected with γ-PGA appear to be encapsulated within a material, suggesting cluster of cells within a γ-PGA matrix. The thickness of γ-PGA coating could be potentially calculated with further testing using transmission electron microscopy (TEM).

### Screening of *B. subtilis* for toxin genes using PCR

**Figure 4:**
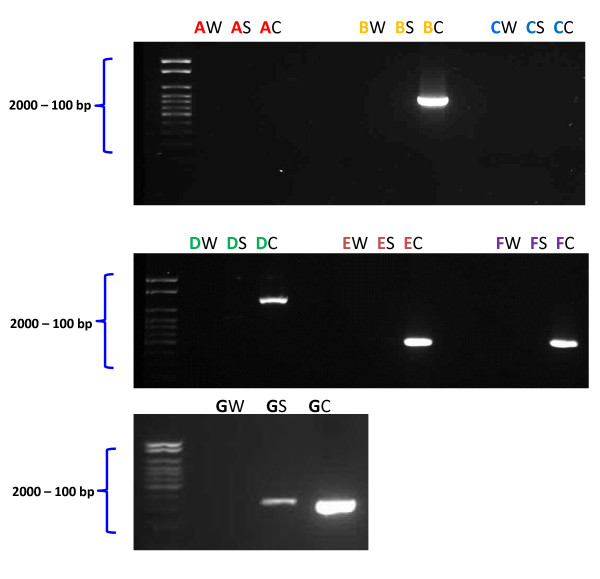
**PCR patterns of *B. subtilis *and *B. cereus *for screening of genes coding for toxins. A** - *hbl*- D/A, **B** – *nheB*, **C** – *bceT*, **D** – *entFM*, **E** – *sph*, **F** – *piplc*, **G** – *pfkA*, W = Water, S = *B. subtilis*, C = *B. cereus.*

The housekeeping gene *pfkA* was seen to be present in both DNA extracts of *B. subtilis* natto and *B. cereus*, confirming successful DNA extraction and PCR amplification (Figure 4). Of the six genes coding for toxin production, four (*nheB*, *entFM*, *sph*, *piplc*) were present in the control organism *B. cereus* (Figure 4). However none of these six genes were present in *B. subtilis* natto. No band was seen in any of the negative control experiments lacking DNA.

### Haemolytic and lecithinase activities of *B. subtilis*

**Figure 5:**
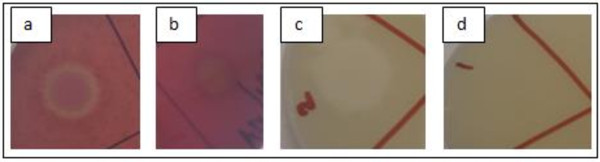
**Physiological analysis of the haemolytic and lecithinase activities of *B. subtilis *natto and *B. cereus*: a – *B. cereus *haemolytic activity showing halo around the cells, b – *B. subtilis *natto haemolytic activity with no halo observed around the cells, c: *B. cereus *lecithinase activity with halo indicating the presence of lecithinase action, d – *B. subtilis *natto lecithinase activity test without halo, indicating the absence of lecithinase activity.**

*B. subtilis* natto and *B. cereus* (positive control) were tested for haemolytic (Figure 5a and b) and lecithinase (Figure 5c & d) activities on blood agar plates and nutrient agar, supplemented with 8% egg yolk emulsion. Large and clear halo formation was seen around the colonies of *B. cereus* on both agars, thus confirming that this bacterium exhibits haemolytic and lecithinase activities (Figure 5). In contrast, no halo formation was seen around the colonies of *B. subtilis* natto, indicating that this bacterium does not exhibit haemolytic or lecithinase activity. The cell-free supernatants of both these bacteria showed similar results.

## Discussion

The antifreeze activity of γ-PGA has been assessed previously (Mitsuiki et al. 1998 Mizuno et al. 1997 Shih et al. 2003). However, it has never been used to maintain and protect the viability of bacteria. This study is the first to assess the effect of γ-PGA on the viability of probiotic bacteria during freeze drying.

On conclusion of the cryoprotectant tests, it was seen that *L. paracasei* was more resilience to the freeze drying process than both the *Bifidobacterium* strains under study, since there was only 1.34 log CFU/ml reduction in viability of unprotected *L. paracasei* when they were freeze dried, whereas the unprotected *Bifidobacterium* strains showed a reduction in viability of around 2.5 log CFU/ml (Figure 2). Wang et al. (2004) found that *Streptococcus thermophilus* and *Lactobacillus acidophilus* exhibited greater survival during freeze drying than did *B. longum* and *B. infantis* (Wang et al. 2004). In contrast, Heidebach et al. (2010) found that probiotic *Bifidobacterium* Bb12 survived better than *Lactobacillus* F19 (Heidebach et al. 2010). Otero et al. (2007) studied the effect of freeze drying on different *Lactobacillus* species and found considerable variation in survival between species and even strains of the same species (Otero et al. 2007). Similar results have been found for members of *Bifidobacterium* species and strains (Lian et al. 2002). Based on the above, it could be concluded that resistance to the freeze drying process varies between species and even strains of the same species. This study demonstrates that *L. paracasei* is more resistant to the freeze drying process than *B. longum and B. breve.*

The results for freeze drying with γ-PGA as a cryoprotectant showed that for *L. paracasei*, sterilised 10% γ-PGA could protect the cells during freeze drying significantly better than 10% sucrose (P < 0.05). Although 5% γ-PGA was also able to protect the cells during freeze drying as efficiently as sucrose (P > 0.05), it was not as efficient as 10% γ-PGA. For *B. longum* and *B. breve*, 10% sterilised γ-PGA and 10% sucrose were equally efficient in maintaining viability during freeze drying (P > 0.05).

It was also observed that the sterilised γ-PGA (obtained by autoclaving an aqueous solution of γ-PGA) was a better cryoprotectant than unsterilised polymer which was initially used for preliminary cryoprotection analysis (results not shown). It has been shown that heating an aqueous solution of γ-PGA can reduce its molecular weight (Goto and Kunioka 1992). Previous studies have also demonstrated that γ-PGA with a lower molecular weight has a higher antifreeze activity than a high molecular weight polymer (Mitsuiki et al. 1998 Shih et al. 2003). This may explain why sterilising γ-PGA enhanced its cryoprotectant ability. FTIR analysis revealed that the structural integrity of the polymer remained intact after steam sterilization (Figure 1). This is in agreement with the study done by Goto and Kunioka (1992) which suggested that the activation energy of the polymer chain scission due to steam sterilization of γ-PGA by heating is approximately 120 kJ/M (Goto and Kunioka 1992). The relative bond strengths of the C-C, C-N and C-O bonds are greater than 300 kJ/M, hence, the breaking of the bonds by heating at 110°C is unexpected.

It has been found that sucrose offers better protection during freeze drying of lactobacilli when compared to trehalose and sorbitol (Siaterlis et al. 2009). Since γ-PGA could protect lactobacilli better than sucrose in this study, it could indicate that γ-PGA is a better cryoprotectant than trehalose and sorbitol as well. *Nata*, a bacterial cellulose produced by *Acetobacter xylinum*, has also been used to protect different lactobacilli during freeze drying (Jagannath et al. 2010). When the cells were immobilized using *nata* and freeze dried, it was observed that viable cell number decreased from 10^9^ – 10^10^ CFU/g to 10^7^ CFU/g. In this study, there was only a 0.51 log CFU/ml and 1.3 log CFU/ml reduction in the viability of γ-PGA-protected *Lactobacilli* and *Bifidobacteria* respectively (Figure 2), indicating that γ-PGA may be a better cryoprotectant compared to *nata*. However, the protection offered by γ-PGA, trehalose, sorbitol and *nata* during freeze drying of *Lactobacilli* needs to be directly compared under identical conditions.

Following the discovery and use of *B. subtilis* natto in the solid state fermentation of soybean to produce the common Japanese food natto, there has been a surge in the industrial application of *Bacillus sp*. for the production of a wide range of useful products (Schallmey et al. 2004). However the detection of toxins and the genes that produce them in some strains of *Bacillus* (Beattie and Williams 1999 Phelps and McKillip 2002) has questioned the use of *Bacillus sp*. in industrial production of several products, especially food and health products. There have been reports where the production of emetic toxins by different *Bacillus sp.*, including *B. subtilis* isolated from food, water and food plants has been demonstrated (From et al. 2005). However, it is important to note that the authors concluded that the tendency of toxin production in strains of *Bacillus* other than *B. cereus* isolated from food, water and food plants is rare. The occurrence of genes capable of producing toxins in other strains of *Bacillus* sp. has already prompted the Scientific Committee on Animal Nutrition (SCAN) to recommend that products from *Bacillus sp*. other than those from *B. cereus* group should be accepted only if there is no detection of toxin production (SCAN 2000). For other products of *Bacillus sp.* which do not include the whole microorganism, it has been recommended that the producing strain should be shown not to produce toxins under production conditions. Therefore, to make γ-PGA applicable in the probiotic food industry as a cryoprotectant, it was crucial to analyse the γ-PGA producing strain for the presence of genes that are known to produce toxins. Moreover, there is presently no report on the toxigenic potential of *Bacillus subtilis* natto, which was used for the production of γ-PGA in our study.

Therefore, the toxigenic potential of *B. subtilis* natto was assessed. The results of the toxigenic analysis in this study showed that none of the six genes known to produce toxins (*hbl D/A*, *nheB*, *bceT*, *entFM*, *sph*, *piplc*) were present in *B. subtilis* natto. Also, physiological analysis showed the absence of haemolytic and lecithinase activities in *B. subtilis* natto. In contrast, the positive control *B. cereus* showed the presence of four of the genes (*nheB, entFM, sph, piplc*) and also exhibited both haemolytic and lecithinase activities. These results are similar to those obtained by (Matarante et al. 2004) who reported the absence of the genes (*hbl D/A*, *nheB*, *bceT*, *entFM*, *sph* and *piplc*) in all the strains of *Bacillus sp.* isolated from sausages and more importantly in *B. subtilis*. Our results are in accordance with another study that investigated the cytotoxic potential of other strains of *B. subtilis, B. licheniformis, B. cereus and B. amyloliquefaciens* used in industrial production of enzyme products and discovered that none of the industrial strains demonstrated any in vitro cytotoxic activity (Pedersen et al. 2002).

In conclusion, this study has established that γ-PGA could be used successfully to improve the survival of probiotic bacteria when during freeze drying. Three probiotic bacteria (*L. paracasei*, *B. longum*, *B. breve*) were successfully protected with γ-PGA when they were freeze dried. It was also seen that *Lactobacillus* was more resistant to the freeze drying process than *Bifidobacteria*. While choosing an agent to protect probiotic bacteria, it is essential to choose one that protects them during the different stages of their production and upon incorporation into food products. Therefore, future work will involve studying the ability of γ-PGA to protect probiotic bacteria during storage in a foodstuff and during passage through the gastrointestinal tract.

For γ-PGA to be used in health and food products, it is important to analyse γ-PGA producing strains for the presence of genes that are known to produce toxins. This research successfully confirmed that *B. subtilis* natto, the bacterium used to produce γ-PGA for the novel application, does not contain any of the genes that are usually responsible for toxin production. In addition, the absence of haemolytic or lecithinase activity in *B. subtilis* natto was also demonstrated.

Therefore, this study suggests that γ-PGA could be used as a food ingredient for the delivery of probiotic bacteria.

## Competing interests

The authors declare that they have no competing interests.
